# Global OMI HCHO Level-3 oversampling dataset: high spatial resolution and lightweight uncertainty

**DOI:** 10.1038/s41597-026-06577-w

**Published:** 2026-01-19

**Authors:** Hui Xia, Dakang Wang, Xiankun Yang, Xicheng Li, Lei Zhu, Tianyu Lu, Zhaolong Song, Yongru Mo, Chenglong Yan, Dongchuan Pu, Xiaoxing Zuo, Wenfu Sun, Jinnian Wang, Xingfa Gu

**Affiliations:** 1 School of Geography and Remote Sensing, Guangzhou University, Guangzhou, 510006 China; 2 Institute of Aerospace Remote Sensing Innovations, Guangzhou University, Guangzhou, 510006 China; 3 School of Environmental Science and Engineering, Southern University of Science and Technology, Shenzhen, 518055 China; 4 College of Science, Northeastern University, Boston, MA02115 USA; 5 School of Architecture & Urban Planning, Shenzhen University, Shenzhen, 518060 China; 6 Royal Netherlands Meteorological Institute (KNMI), De Bilt, the Netherlands; 7 Department of Geoscience & Remote Sensing, Delft University of Technology (TUD), Delft, the Netherlands; 8 Division of Atmospheric Composition, Royal Belgian Institute for Space Aeronomy (BIRAIASB), Brussels, 1180 Belgium

**Keywords:** Environmental impact, Atmospheric dynamics

## Abstract

Satellite observations of tropospheric formaldehyde (HCHO) have been widely used to support diagnosing atmospheric environmental quality. As one of the most classic trace gas payloads, the Ozone Monitoring Instrument (OMI) releases HCHO Level-2 data, while coarse resolution and relatively high uncertainty reduce the potential value of the data. We report a global multi-scale HCHO oversampling dataset version 1.0 (referred to as OMHCHOS V1.0) produced by NASA Level-2 OMI HCHO product using self-developed oversampling algorithm, with data from 2005 to 2023 as of the date of submission. This comprehensive dataset encompasses seven distinct spatial resolutions (up to 0.05°) and twelve temporal resolutions (monthly and months), enabling precise quantification of uncertainty propagation and relative uncertainties. To facilitate on-demand retrieval by users, we have developed a matching spatio-temporal scale optimisation model that integrates three critical parameters of HCHO column: temporal resolution (TR), spatial resolution (SR), and relative uncertainty (UR). This dataset will provide researchers with more reliable sources for conducting high-resolution, high-accuracy studies on HCHO-related atmospheric environmental implications.

## Background & Summary

Atmospheric formaldehyde (HCHO) is a short-lived trace gas with toxicity and carcinogenicity^1,2^. As a key participant in numerous photochemical processes, it significantly influences ozone (O_3_) formation and contributes to the generation of secondary organic aerosols (SOA)^3–5^. In the context of air pollution control, HCHO serves as a useful proxy for volatile organic compounds (VOCs), helping to indicate their spatiotemporal distribution and behavior^6,7^, which contributes to explore the deeper mechanisms of atmospheric component interactions, long time-series and large-scale accurate tracing for atmospheric HCHO is of significant research value.

In recent years, the international scientific community has relied on HCHO products from multiple satellite missions, including the GOME series (40 km × 8 km ~ 320 km × 40 km), SCIAMACHY (120 km × 30 km), OMPS series (17 km × 17 km ~ 50 km × 50 km), OMI (13 km × 24 km), TROPOMI (3.5 km × 7 km), GEMS (3.5 km × 8 km), and TEMPO (2 km × 4.75 km). The Ozone Monitoring Instrument (OMI) payload, launched in 2004, represents a landmark achievement in atmospheric monitoring and remains operational as the longest-serving trace gas observation satellite. With products continuously updated for over two decades, OMI has provided an luxuriant global dataset for HCHO satellite observation^8^. TROPOMI has achieved kilometer-scale spatial resolution, while publishes short time series^9,10^, GEMS and TEMPO do not support global observation^11–13^. OMI HCHO data remain the best candidate to support global long time series monitoring of atmospheric HCHO. Fan *et al*. (2021) revealed the spatial and temporal distribution of HCHO in the east coast of China from 2009-2018 using OMI HCHO observations^14^. Shen *et al*. (2019) evaluated long-term trends in emission inventories of volatile organic compounds (VOCs) that affect air quality using OMI HCHO data^15^. Wang *et al*. (2022) contrasted the differences between OMI observations and ground-based monitoring data in the United States from 2006 to 2015 and diagnosed the seasonal variation of formaldehyde emissions in US^16^. Previous researches predominantly processed Level-2 (L2) OMI HCHO data at relatively coarse spatial resolutions (dozens of kilometers), many pixels are discarded because of their original pixel-level uncertainties during data screening, causing gaps or incomplete regional coverage, and it is not uncommon to not even consider the uncertainty of the original L2 data. OMI HCHO data have provided invaluable support for atmospheric research, and achieving high spatial resolution with lightweight uncertainty would better meet the demands of precise and comprehensive analysis.

EARTHDATA has released daily L2 OMI HCHO data (13 km × 24 km) and gridded daily L3 data (updated to June 2022 at 0.1° resolution when writing this manuscript) with detailed data algorithms and explanatory documents. Although a significant improvement over earlier products, the spatial resolution of OMI L2 HCHO data remains insufficient for pinpointing fine-scale emission sources at the kilometer level. The coarser-resolution L3 data offer limited temporal coverage and are primarily suitable for large-scale emission analysis. As noted in the OMI HCHO product documentation, attributing to the propagation and accumulation of multiple process uncertainties during the retrieval algorithm, the comprehensive uncertainty of L2 HCHO vertical column density (VCD) gives 50% to 105%^17^, which brings negative speculation on the reliability of the study conclusions. The L2 HCHO data published by EARTHDATA are single-orbit data at transit time, 14–15 orbits per day to achieve global coverage, research on 20-year global atmospheric HCHO monitoring will have to deal with more than 100,000 L2 files, which costs a lot of time and arithmetic. Researchers commonly apply mathematical interpolation to generate gridded datasets^18,19^, while interpolation methods frequently fail to account for the spatial heterogeneity of both the surface and the atmosphere.

Global OMI tropospheric HCHO gridded data with high spatial resolution, low uncertainty level and easy accessibility show great demand. We constructed a multi-scale, long time sequence global OMI HCHO L3 oversampling dataset (OMHCHOS) using an independently developed oversampling algorithm. This approach fully leverages the spatial distribution information from multi-temporal trace gas satellite observations, enhancing the spatial resolution of trace gas retrievals to the kilometer scale, to enable high-precision identification of pollution and emission sources and is freely available to scientific community. OMHCHOS data exhibits several features compared to the L2 HCHO product provided by EARTHDATA: (I) The spatial resolution has been refined from the original tens of kilometers to the kilometer scale (0.05°), substantially improving the capability to monitor HCHO emission sources at fine spatial scales. (II) Each grid contains high-resolution HCHO VCD along with integrated uncertainty information propagated from the original L2 data. Users can selectively utilize the data based on their specific uncertainty requirements, such as determining relative uncertainties below 0.25, while still ensuring abundant data available for use. (III) OMHCHOS expand the data dimensions to provide multi-scale spatial and temporal resolution, seven spatial resolutions are built, including 0.05°, 0.1°, 0.2°, 0.3°, 0.5°, 0.75°, and 1.0°, and twelve temporal resolutions, spanning from 1 month to 12 months, so that users select data according to their needs. (IV) To facilitate efficient data retrieval, OMHCHOS include a matching spatio-temporal scale optimisation model, which enables intuitive visualization of dataset characteristics, promoting users to determine the optimal data download scheme quickly and effectively. As of the submission date, OMHCHOS covers the period from 2005 to 2023 and will be continuously updated. OMHCHOS is freely available to researchers worldwide, providing a richer, more accessible, and more reliable dataset for atmospheric HCHO explorations.

## Methods

The OMHCHOS V1.0 dataset was produced by applying our self-developed oversampling algorithm to L2 OMI HCHO products, we processed a total of 95782 orbits of OMI HCHO L2 data from 2005–2023 (with volume of 2.6 TB). By enabling flexible specification of grid spatial resolution, the algorithm effectively leverages the spatial distribution information inherent in multi-temporal satellite L2 pixel observations of trace gases. This approach enhances the spatial resolution of trace gas satellite remote sensing products to the kilometer scale while simultaneously reducing the uncertainty levels introduced by the original satellite measurements. The core principle involves accounting for the spatial variation in the satellite detector’s response to radiation within a single L2 pixel, characterized by the detector’s spatial response function (SRF), which typically follows a bivariate Gaussian distribution, where the signal is strongest near the pixel center and weakest at its boundaries. Exploiting the spatial overlap of satellite L2 pixels across different overpasses, the oversampling algorithm combines data from multiple time periods to generate gridded data with a higher resolution than the native satellite data. During the gridding process, the uncertainty associated with the original observations undergoes transformation^20,21^. We derived an uncertainty propagation formula for the oversampling algorithm based on the law of linear uncertainty propagation. This formula quantitatively describes the evolution of uncertainty from the initial satellite measurements through the functional transformations involved in the algorithm (e.g., oversampling). The propagated uncertainty and relative uncertainty metrics are crucial for subsequent data quality control and visualization noise filtering, enhancing the reliability of the final product. Test and validation results demonstrate that the algorithm operates efficiently. It fully utilizes multi-temporal information, effectively balances pixel size and spatial response function considerations, and produces gridded column concentration distributions with higher signal-to-noise ratios and improved spatial resolution. This capability enables superior identification of regions with elevated trace gas column density. Furthermore, the algorithm effectively filters noise and anomalous data, yielding more reliable oversampling results and spatial mapping^22^.

The oversampling algorithm operates by assigning a predefined spatial resolution to a given grid $i$, intersecting with satellite pixel $p$, whose HCHO VCD is represented as $\Omega (p)$. The superimposed area between the grid $i$ and the pixel $p$ is denoted as $A(p,i)$, the contribution weight of the HCHO signal from one satellite pixel is determined by the ratio of $A(p,i)$ and the area of the satellite pixel $S(p)$. With the continuous superimposition of multiple orbits, there will be multiple pixels contributing to the HCHO signal, and the final HCHO VCD for each grid, derived through the oversampling algorithm, is expressed in Eq. (1). This algorithm enables flexible customization of the spatial resolution and coverage capacity of OMI HCHO data while effectively reducing the uncertainty in the spatial distribution of formaldehyde.

1 $$\bar{\varOmega }(i)=\frac{{\sum }_{p=1}^{N(i)}\frac{A(p,i)}{S(p)}\Omega (p)}{{\sum }_{p=1}^{N(i)}\frac{A(p,i)}{S(p)}}$$

The uncertainty of the *p*-th pixel is ${\rm{\sigma }}({\rm{p}})$, according to the uncertainty propagation rule, and the composite uncertainty of the grid obtained by oversampling is expressed in Eq. (2). This dataset further quantitatively tracks the evolution of uncertainty to assess the capability of oversampling algorithm in diminishing the uncertainty level of original Level-2 formaldehyde.

2 $$\sigma (i)=\sqrt{\frac{{\sum }_{p=1}^{N(i)}{\left[\frac{A(p,i)}{S(p)}\cdot \sigma (p)\right]}^{2}}{{\left[{\sum }_{p=1}^{N(i)}\frac{A(p,i)}{S(p)}\right]}^{2}}}$$

**Fig. 1:**
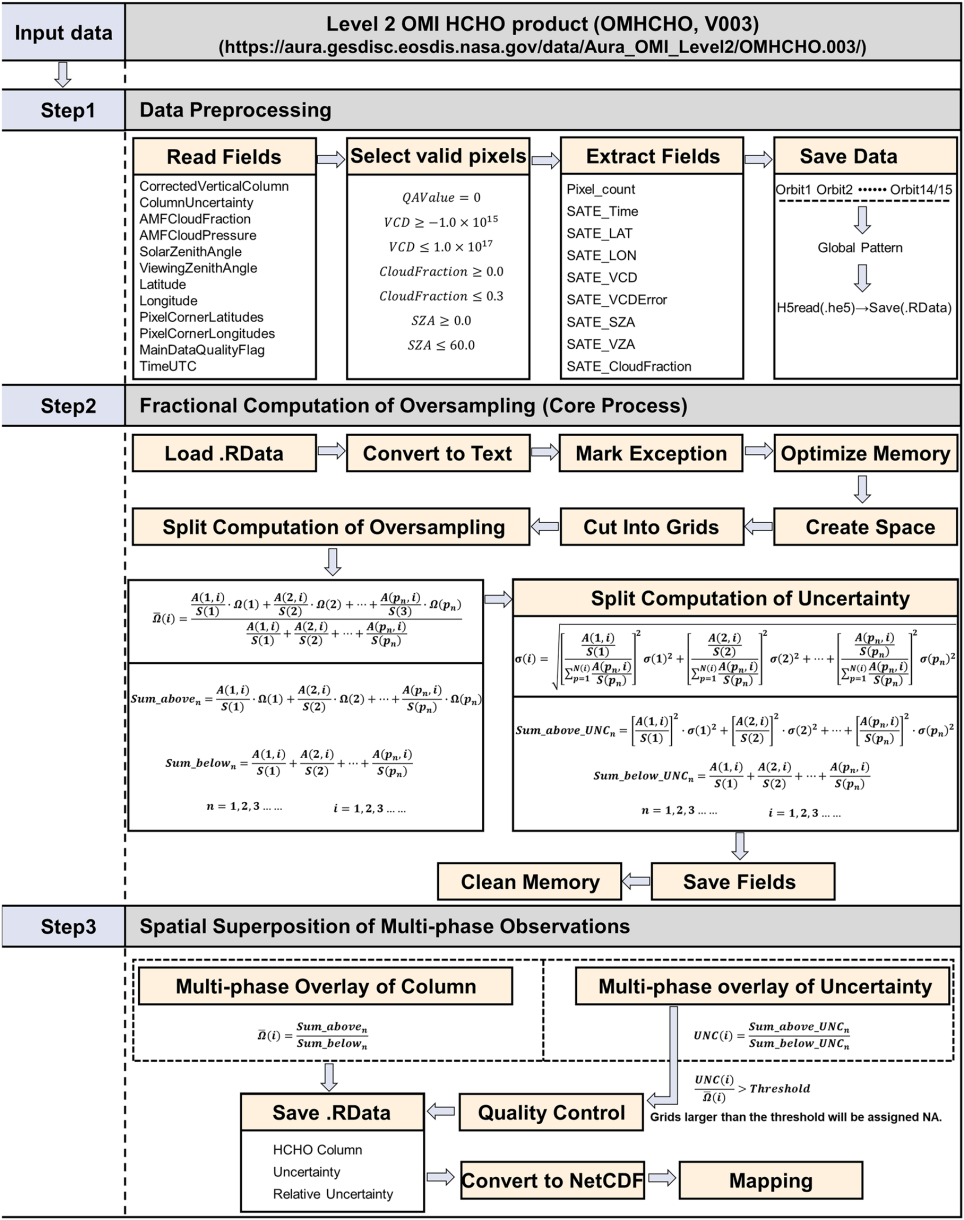
Overall working framework for producing OMHCHOS V1.0 dataset from L2 OMI HCHO observations.

Figure 1 illustrates the workflow of the oversampling algorithm, which comprises three principal steps. Step one is data preprocessing. Step two is fractional computation of oversampling, and the final step is spatial superposition of multi-phase observations.

### Input datasets

The L2 OMI HCHO observations served as the foundational source for generating the L3 gridded OMHCHOS data, which is freely available from NASA (https://aura.gesdisc.eosdis.nasa.gov/data/Aura_OMI_Level2/OMHCHO.003/). OMI was launched aboard NASA’s Earth Observing System (EOS) Aura satellite on 15 July 2004 and has been continuously collecting data since 9 August 2004. The instrument features a nadir spatial resolution of 13 km × 24 km and operates within a spectral range of 264–504 nm. With a swath width of 2,600 km, OMI achieves near-global coverage through 14–15 orbits per day. The L2 OMI HCHO data are stored in HDF-EOS format, with each file containing a daily observation over a single orbit. The L2 OMI HCHO products are released by the Smithsonian Astrophysical Observatory (SAO), which extracts backscattered radiation in the solar ultraviolet band to retrieve atmospheric formaldehyde according to the absorption characteristics of formaldehyde spectrum. This process involves three key steps: (I) Determination of the slant column density (SCD) through spectral fitting using the Basic Optical Absorption Spectroscopy (BOAS) method, with a fitting window of 328.5–356.5 nm; (II) Conversion of SCD to VCD via the atmospheric mass factor (AMF) computed using a radiative transfer model; and (III) Post-processing trans-orbital deviation corrections, to generate the final OMI HCHO product^18^. OMI HCHO Level 2 data (V003) spanning the period from 1 January 2005 to 1 October 2023 were utilized for this research.

### Algorithm execution

The core implementation of the oversampling algorithm is developed in Fortran. We invoke Fortran programs, along with supplementary Shell scripts, via R language interface APIs. The entire workflow is engineered to seamlessly execute within the RStudio environment through three distinct computational stages.

**Step1:** Data Preprocessing

The algorithm execution begins with the cyclic reading and conditional filtering of multi-orbit data using R interface. Pixels that pass the quality check will be extracted into a vector, and then the valid pixels will be placed into a global pattern matrix formed by integrating orbits when the statistical process is completed. Quality control parameters set during this process include defining HCHO VCD thresholds (−1 × 10^15^ molec/cm^2^, 1 × 10^17^ molec/cm^2^), cloud fractions (0–0.3), and solar zenith angle (0–60°), and rows from 1–20 and 55–60 of the OMI detector were selected given that row anomaly^23^, contribution of high-quality pixels will continue to improve the numerical accuracy of the grid.

**Step2:** Fractional Computation of Oversampling

The fractional computation of oversampling and uncertainty propagation are performed at predefined grid resolutions, which is the core computational procedure of the oversampling algorithm. High-quality data acquired from Step1 are loaded, and futher converted to text for anomaly labeling, and subsequently compressed. This compressed text serves as the primary input for the Fortran program, which will be transferred to a designated temporary workspace where the source program is invoked. Within this environment, critical operations are performed including efficient data slicing along both longitudinal and latitudinal directions, computation of the vertical column density ($\bar{\varOmega }(i)$) fraction components, and the uncertainty ($\sigma (i)$) fraction components. This design specifically stores the numerator and denominator terms from Eqs. 1 and 2 individually, substantially enhancing computational and storage efficiency.

**Step3:** Spatial Superposition of Multi-phase Observations

At a prescribed spatial resolution, the spatial superposition of multi-phase grids is performed. Specifically, the ratio of the numerator and denominator obtained in Step 2 is calculated to derive the final values for vertical column density ($\bar{\varOmega }(i)$) and its associated uncertainty ($\sigma (i)$). A threshold is applied to the relative uncertainty, grid values exceeding this threshold are assigned NoData, ensuring the generation of a high-quality Level 3 product, which is subsequently output in the widely accepted NetCDF format for downstream processing and mapping. During producing the dataset, the gridded spatial resolution was set to 0.05°, 0.1°, 0.2°, 0.3°, 0.5°, 0.75° to 1.0°, and the temporal resolution was set from 1 to 12 months.

### Satellite-based validation datasets

The Level-3 daily HCHO product (OMHCHOd) released by EARTHDATA serves as the validation data, spatial resolution is 0.1° × 0.1°, and are stored in netCDF4 format, with each file containing daily global HCHO observation. The HCHO VCD in the dataset represent the weighted average of cloud-screened daily HCHO VCD within each grid cell^19^. The temporal coverage of OMHCHOd spans from 1 October 2004 to 23 June 2022.

### MAX-DOAS validation datasets

The QA4ECV MAX-DOAS HCHO product was jointly developed by the Royal Belgian Institute for Space Aeronomy (BIRA-IASB) and collaborating teams within the framework of the EU-FP7 QA4ECV project^24,25^. The raw spectra collected from multiple observational sites were retrieved using a harmonized algorithm, producing rigorously quality-controlled and standardized HCHO columns, which were retrieved with two fitting windows: 324.6–359 nm and 336.5–359 nm. The product includes metadata such as instrument operation start time, HCHO total tropospheric column, total random uncertainty, and total systematic uncertainty. MAX-DOAS datasets serve as ground-based observations to validate and evaluate the oversampling dataset.

### GEOS-Chem validation datasets

GEOS-Chem is a global three-dimensional chemical transport model (CTM) designed to simulate the distribution, transport, and deposition of atmospheric constituents. Developed by NASA Global Modeling and Assimilation Office (GMAO), GEOS-Chem utilizes meteorological assimilation data as driving force to model atmospheric sources, sinks, and various physicochemical processes. Renowned for its accuracy and versatility, GEOS-Chem has been widely adopted in atmospheric research across a broad range of applications^26–28^. This research utilized GEOS-Chem version 12.9.3, driven by MERRA-2 meteorological data, with a spatial resolution of 2° × 2.5° and 47 vertical layers. Anthropogenic emissions were derived from the Community Emissions Data System (CEDS), biomass burning emissions were sourced from the Global Fire Emissions Database (GFED v4), biogenic emissions were calculated online using the Model of Emissions of Gases and Aerosols from Nature (MEGAN v2.1). The simulation spin-up period spanned from June 2017 to May 2023, with the final simulation covering June 2018 to April 2023, ensuring the assessment of data quality over the last five years within the valid timeframe of the oversampling dataset. TROPOMI averaging kernels were applied for calibration to refine the GEOS-Chem simulation. Statistical results confirmed a strong linear relationship between the simulated and observed HCHO VCD^29^, validating the dataset for further atmospheric research applications.

### Oversampling spatio-temporal scale optimisation model

We constructed an oversampling spatio-temporal scale optimisation model (OSTSOM) that integrates three critical dimensions: temporal resolution (TR), spatial resolution (SR), and relative uncertainty (UR) of HCHO VCD, aiming to systematically visualize the overall characteristics and evolution patterns of the OMI L3 HCHO oversampling dataset, allowing users to quickly determine the optimal oversampling data selection scheme based on their needs for the dataset. Given any two of the three parameters, OSTSOM expeditiously tells users the evolution of the third, providing data selection recommendations tailored to specific research needs. We systematically calculated UR for all oversampling gridded products, deriving global averages to establish the ER axis of OSTSOM, resulting in a total of 1492 average relative uncertainty data. Each point contains four attributes: year, spatial resolution, temporal resolution and average relative uncertainty. Temporal resolution (in months) is represented on the X-axis, spatial resolution (in degrees) on the Y-axis, and relative uncertainty (dimensionless) on the Z-axis. We employed a Rational 2D fitting model (Eq. 3) to perform a nonlinear surface fit across all data points without distinction, achieving a coefficient of determination (R²) of 0.817. Integrate all data points to construct a 3D model and get OSTSOM.

3 $${\rm{z}}({\rm{x}},{\rm{y}})=\frac{{{\rm{z}}}_{0}+{{\rm{A}}}_{01}{\rm{x}}+{{\rm{B}}}_{01}{\rm{y}}+{{\rm{B}}}_{02}{{\rm{y}}}^{2}+{{\rm{B}}}_{03}{{\rm{y}}}^{3}}{1+{{\rm{A}}}_{1}{\rm{x}}+{{\rm{A}}}_{2}{{\rm{x}}}^{2}+{{\rm{A}}}_{3}{{\rm{x}}}^{2}+{{\rm{B}}}_{1}{\rm{y}}{+{\rm{B}}}_{2}{{\rm{y}}}^{2}}$$

## Data Records

**Fig. 2:**
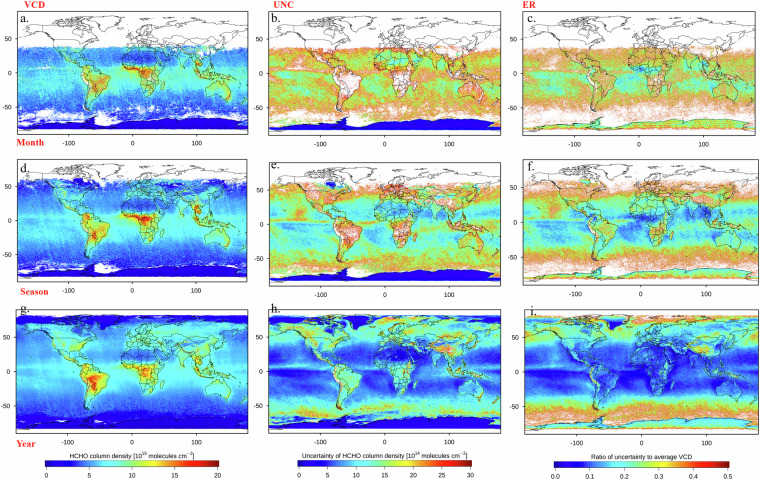
Samples presentation of oversampling dataset (in 2020). The first column is HCHO VCD, the second column is uncertainty, the third column is relative uncertainty. Panels (a-c) are monthly oversampling data, panels (d-f) are quarterly oversampling data, and panels (g-i) are annual oversampling data.

Our OMHCHOS V1.0 dataset is available on the Science Data Bank repository (10.57760/sciencedb.29626)^30^. The total dataset size is approximately 2.3 TB, comprising 18,518 OMI HCHO Level-3 grid products (about 1.6 TB) and their corresponding 18,518 NetCDF files (about 635 GB), along with spatial mapping products (Fig. 2) and relevant oversampling algorithm scripts.

The OMI HCHO Level-3 grid products are the initial gridded datasets generated using the oversampling algorithm in RData format, stored in the ‘*grid_data’* directory. To enhance usability, we also provide a NetCDF4 version with CF-compliant attributes, stored in the ‘*nc_data’* directory. Both the RData and NetCDF datasets follow the same directory structure: they are split into first-level catalogues by spatial resolution, and within each, a second-level catalogue is organized by temporal resolution. These two data types share identical variables and attributes; for example, the gridded data file are named ‘*OMI_HCHO_Global_yyyyy-mm-dd_yyyyy-mm-dd_Res_n.nn_PL_5.RData’*, where *yyyyy-mm-dd* is the time range of the data, *n.nn* is the specific spatial resolution, and *PL* indicates the minimum number of effective pixels in a grid, which is uniformly set to 5 in this dataset, *RData* is the binary file format for data in R. The data contains three fields, *Average_grids*, *Average_UNC_grids* and *UNC_to_Average*, which represent the HCHO VCD, uncertainty and relative uncertainty, respectively. The above storage rules also apply to netCDF files. To enable users to quickly browse and assess data quality, we include visualized images corresponding to the HCHO VCD data. Spatial mappings are stored within the ‘*figs’* directory, organized under secondary directory corresponding to different temporal resolutions, each category includes three graphical representations: the spatial distribution of HCHO VCD, with the name prefix *‘OMI_HCHO_’*, uncertainty, with the name prefix ‘*OMI_HCHO_uncertainty_’*, and relative uncertainty, with the name prefix ‘*OMI_HCHO_uncertainty2average_’*.

In addition to the data, we also provide the core code for executing the oversampling algorithm along with related explanatory files. In the ‘*Codes’* folder, three types of code files are included: ‘*cakecut_src’*, which contains the core oversampling kernel scripts; ‘*code_oversampling’*, which provides the R external interface code; and ‘*code_application’*, which offers example scripts for processing the OMHCHOS V1.0 dataset. Detailed information on the purpose of each script and the system requirements can be found in the ‘*README.txt*’ file. Furthermore, comprehensive explanations of the OMHCHOS V1.0 dataset, including file organization, variable definitions, and data provenance, are available in the ‘*Oversampling_Dataset_Description.json’* sidecar file.

When downloading the data, please enter the full dataset name “Global OMI HCHO Level 3 Oversampling Dataset” in the search bar on ScienceDB to access the download link. All the data are published free of charge and open access to facilitate in-depth exploration for research on tropospheric HCHO. Available for references, numerous air quality researches has been supported by this dataset or algorithm^31–37^.

## Technical Validation

### Comparison with Level 3 OMI HCHO product

We conducted a cross-validation for the oversampling dataset using L3 OMI HCHO product (OMHCHOd) published by EARTHDATA, evaluating data quality in terms of VCD, as well as spatial and temporal distributions. Daily OMHCHOd data were aggregated into monthly composites for convenient comparison. We collected almost all OMHCHOd data (public data only covers January 1, 2005 to June 23, 2022), which were processed into 1-month (monthly), 3-month (quarterly), and 12-month (annual) composites, with VCD constrained within the range of −1 × 10¹⁵ to 1 × 10¹⁷ molec/cm². Effective pixels superimposed into each grid are accumulated and counted, and finally obtain OMHCHOd L3 monthly synthetic data (OMHCHOms) at various time intervals. We extracted oversampling data with spatial resolution of 0.1° and temporal resolutions of 1 month, 3 months, and 12 months, ensuring alignment with the OMHCHOms dataset for consistency.

**Fig. 3:**
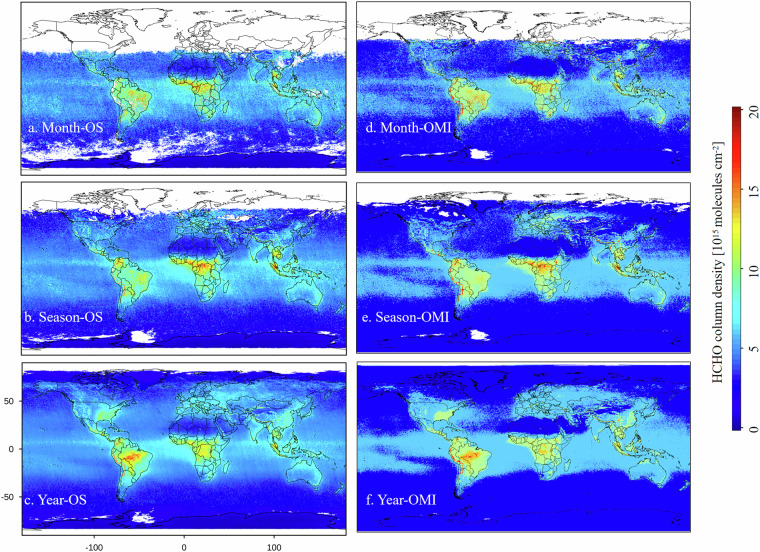
Comparison between OMHCHOS and OMHCHOms (in 2005).

Figure 3 presents a spatial comparison between the oversampling dataset and OMHCHOms, with 2005 as an example. Panels (a–c) illustrate the spatial distribution of the oversampling dataset at monthly (Month-OS), quarterly (Season-OS), and annual (Year-OS) scales, while panels (d–f) depict the spatial distribution of OMHCHOms at monthly (Month-OMI), quarterly (Season-OMI), and annual (Year-OMI) scales. Both give relatively consistent spatial mapping for global scale at different temporal resolutions. High HCHO VCD are primarily located in central South America, central Africa, and Southeast Asia, whereas low HCHO VCD are concentrated in North Africa, the Arctic Ocean, and Antarctica. Additionally, Atlantic Ocean, Indian Ocean, and central Pacific Ocean show large areas of low HCHO VCD, approaching background levels.

**Fig. 4:**
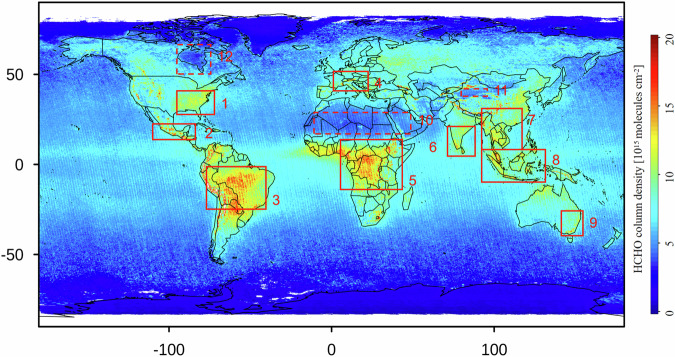
Location of the 12 target regions, with the solid red box showing high HCHO emission and the dashed red box revealing the HCHO emission.

To ensure a geographically unbiased evaluation of the dataset, we also conducted assessment at both large regional scales and localized high and low HCHO VCD regions. On the large scale, we selected South America, Africa, and China; while on the smaller scale, we identified 12 target regions, including 9 high-emission and 3 low-emission regions, to appraise the ability of oversampling dataset for capturing high and low values of HCHO VCD. Target regions are depicted in Fig. 4, where the red solid-line boxes present high-emission, and the red dashed-line boxes show low-emission. Correlation test, error analysis and bias analysis were performed between the oversampling dataset and OMHCHOms at the unit of grid, all the statistical results were classified as years.

**Fig. 5:**
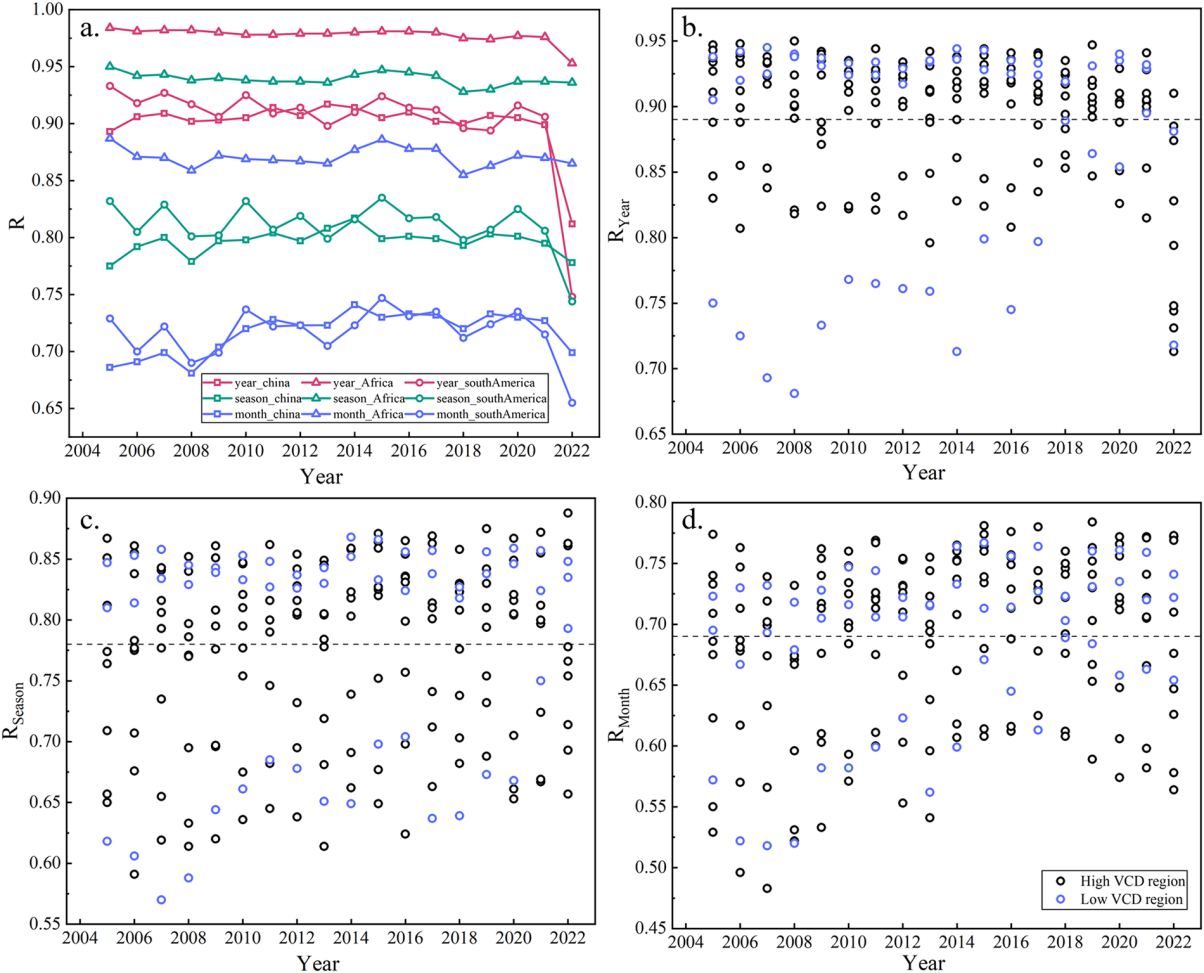
Correlation between OMHCHOS and OMHCHOms for large and small regions. Panel (a) presents the correlation for large regions, where the red line refers to annual scale, the green line represents seasonal scale, and the blue line is monthly scale. Panels (b-d) show the correlation for small regions on annual, quarterly, and monthly scales, where the black circles on behalf of regions with high HCHO emissions and the blue circles present regions with low HCHO emissions.

Figure 5 shows significant correlation between OMHCHOS and OMHCHOms for both small and large regions. Figure 5a presents the correlation at three temporal resolutions for large-scale regions, with mean correlation coefficients (*R*) are 0.93 (annual), 0.85 (quarterly), and 0.77 (monthly), all of which are statistically significant (*p* < 0.05), the larger the temporal resolution, the better the correlation, implying that the more contribution from effective pixels will further improve the accuracy of the oversampling dataset. Africa exhibited the highest correlation (*R* = 0.93), while South America (*R* = 0.81) and China (*R* = 0.80) are close. Panels (b–d) illustrate the correlation between the two datasets at small regional scales over annual, quarterly, and monthly periods, respectively. The average *R* for all regions is 0.89 (annual), 0.78 (quarterly), and 0.69 (monthly), which was marked by a dashed line in the panel. The high and low emission regions of HCHO can be found at both above and below the average value, demonstrating the weak influence to the correlation between the two datasets from the level of HCHO VCD. According to the comparison about large and small regions, correlation of the large region is slightly better than that of small region, but with no significant difference, which suggests stable and reliable quality of oversampling dataset, regardless of regional scale.

**Fig. 6:**
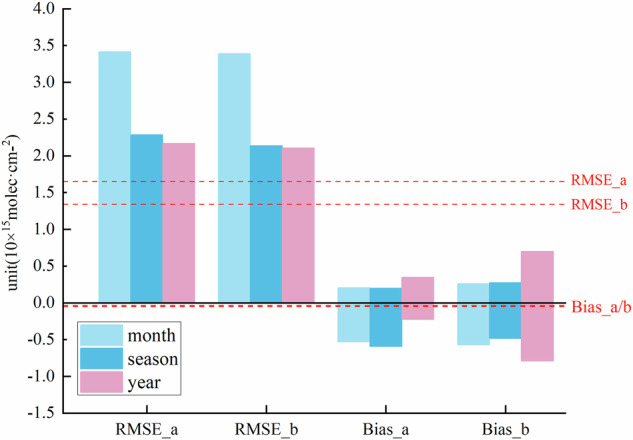
RMSE and bias for OMHCHOS with OMHCHOms in large and small areas. ‘a’ represents large region, ‘b’ refers to small region. The red dashed line marks the average value for all regions. Average bias for large and small region is plotted as a single line because of their similarity.

Root mean square error (RMSE) and bias were employed to assess the quality of the oversampling datasets. RMSE quantifies the magnitude of mean error between the two datasets, smaller values prove reduced discrepancies, while bias judges the degree of underestimation or overestimation between datasets, with smaller values suggesting minimal deviation. Johnson *et al*. (2022) averaged the spatial resolution of both quarterly HCHO data captured by the airborne sensors and satellite data to 0.15° × 0.15°, and then the airborne HCHO data was compared and validated against OMI HCHO and QA4ECV OMI HCHO, yielding an average RMSE of approximately 9.0 × 10^15^ molec/cm^2^, and the biases were found to be approximately 5.1 × 10^15^ molec/cm^2^ and 2.3 × 10^15^ molec/cm^2^, respectively^38^. Liao *et al*. (2025) reported the average biases between NASA ATom in situ HCHO observations and OMI SAO, OMPS SAO observations in oceanic regions across seasons, with values of (−0.73 ± 0.87) × 10^15^ molec/cm^2^ and (−0.76 ± 0.88) × 10^15^ molec/cm^2^, respectively^39^. We computed the average RMSE and bias for both large and small regions across three temporal resolutions (monthly, quarterly, and annually), as shown in Fig. 6, which unveils lower RMSE and bias of our dataset than those reported by Johnson *et al*. (2022), and close to the values given by Liao *et al*. (2025).

The bar charts in Fig. 6 display the RMSE and bias for all data in the two types of regions. RMSE of oversampling dataset is close for both large and small regions. There is little difference between RMSE at quarterly and annual level, with the largest RMSE at the time resolution of monthly, which is about 3.5 × 10^15^ molec/cm^2^. Bias of the oversampling dataset fluctuates around zero, monthly bias is close to quarterly, with the ranges of about (−0.5 × 10^15^ molec/cm^2^, 0.2 × 10^15^ molec/cm^2^). Bias for the annual dataset in small regions is a bit pronounced, ranging from (−0.8 × 10^15^ molec/cm^2^, 0.7 × 10^15^ molec/cm^2^). The average RMSE for the large and small regions are 1.66 × 10^15^ molec/cm^2^ and 1.35 × 10^15^ molec/cm^2^, respectively, with average biases of −0.04 × 10^15^ molec/cm^2^ and −0.05 × 10^15^ molec/cm^2^. To sum up, the evaluation indicates relatively low RMSE and bias.

### Comparison with MAX-DOAS data

In this study, the QA4ECV MAX-DOAS dataset was adopted as an independent ground-based reference for validation. We specifically employed station data retrieved using the 324.6–359 nm fitting window and selected three representative sites: Xianghe, Uccle, and Mainz, covering both suburban and urban environments (Table 1). We compiled the HCHO total tropospheric columns from each site over different temporal ranges. Following the validation strategy proposed by Isabelle De Smedt^40^, daily averages were obtained by averaging all MAX-DOAS retrievals between 11:00 and 16:00 local time, and monthly means were then derived from the available daily observations.

**Table 1:** MAX-DOAS stations information.

Site	Lat, Long	Class	Data Source	Time coverage
Xianghe	39°N, 117°E	Sub-urban	BIRA	04/2010–01/2017
Uccle	50°N, 4°E	Urban	BIRA	04/2011–06/2015
Mainz	50°N, 8°E	Urban	MPIC	06/2013–12/2015

**Fig. 7:**
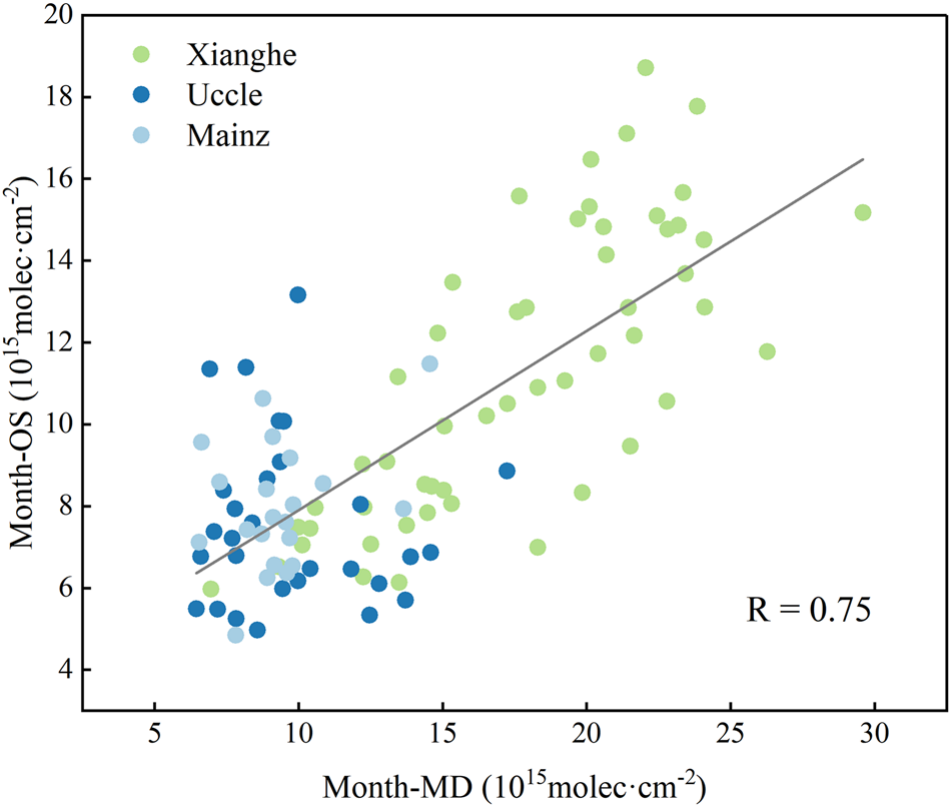
Scatter plots of OMHCHOS versus MAX-DOAS Observations in Xianghe, Uccle and Mainz stations.

For comparison, we utilized OMHCHOS with a spatial resolution of 0.05° and a monthly temporal resolution. Based on the geolocation of the MAX-DOAS sites, the selected OMHCHOS were extracted within a 20 km radius, and the corresponding monthly averages were calculated for the three representative sites. Validation results (Fig. 7) show that OMHCHOS and the ground-based observations exhibit highly consistent temporal patterns (R = 0.75). RMSE is within a reasonable range (3.87 × 10¹⁵ molec/cm²), and the bias (−4.21 × 10¹⁵ molec/cm²) indicate a slight systematic underestimation in OMHCHOS. This bias is consistent across cases and can be readily corrected.

### Comparison with simulation data

**Fig. 8:**
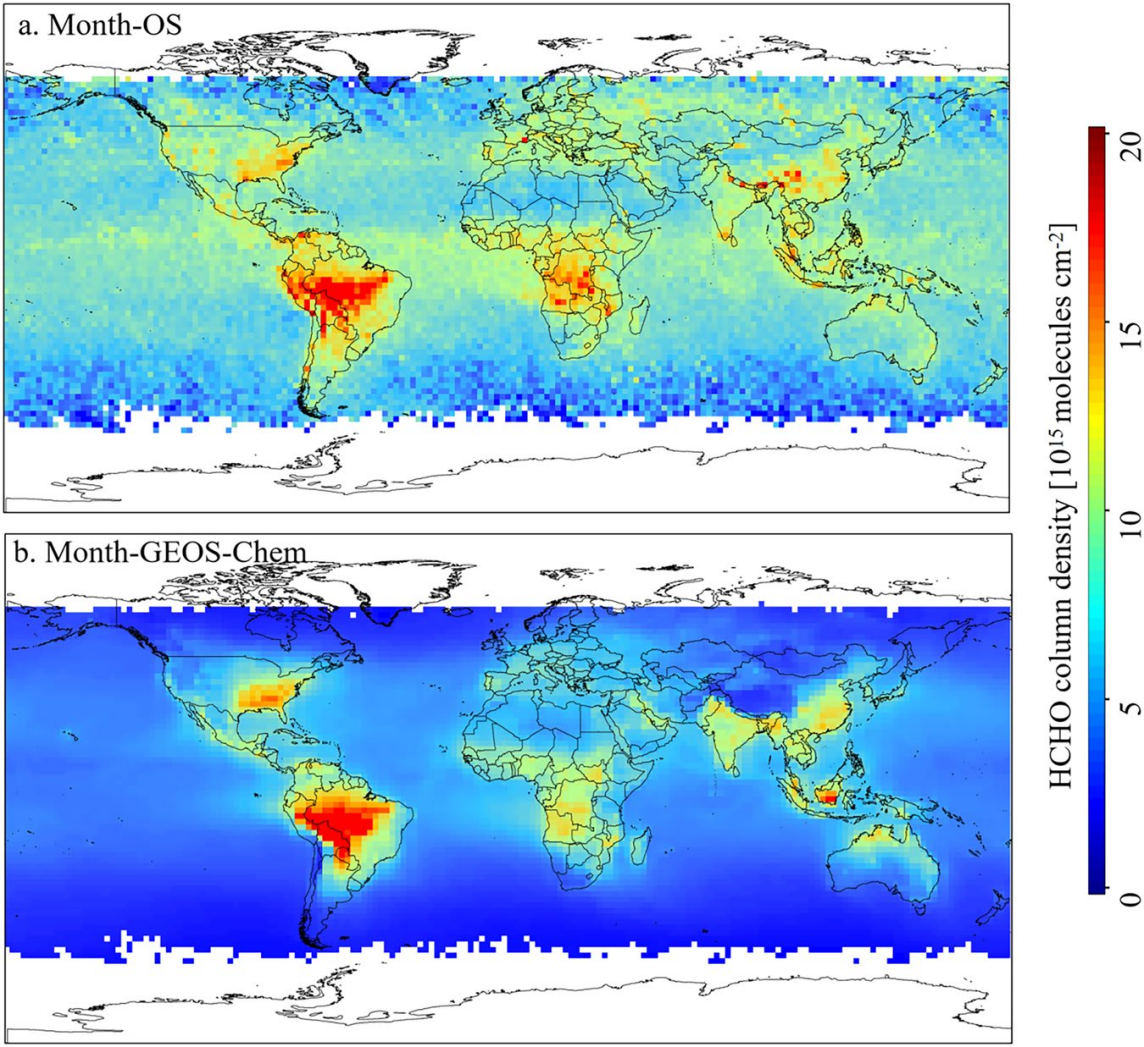
Comparison of oversampling dataset with GEOS-Chem simulated data at 2° spatial resolution (September 2018).

The GEOS-Chem simulated data and the oversampling dataset, with spatial resolution of 1° and time range from June 2018 to April 2023, are resampled to a 2° resolution, with temporal resolution on a monthly scale. Figure 8 presents the spatial distribution of oversampling dataset and simulated data, the global mapping from oversampling dataset is generally consistent with simulated data. Both data show that high HCHO VCD occur in central South America, southeastern North America, central Africa, and Southeast Asia, which testifies their consistency in monitoring the global distribution of high and low HCHO emission areas, capturing regions of high HCHO emissions is the priority of HCHO monitoring.

**Fig. 9:**
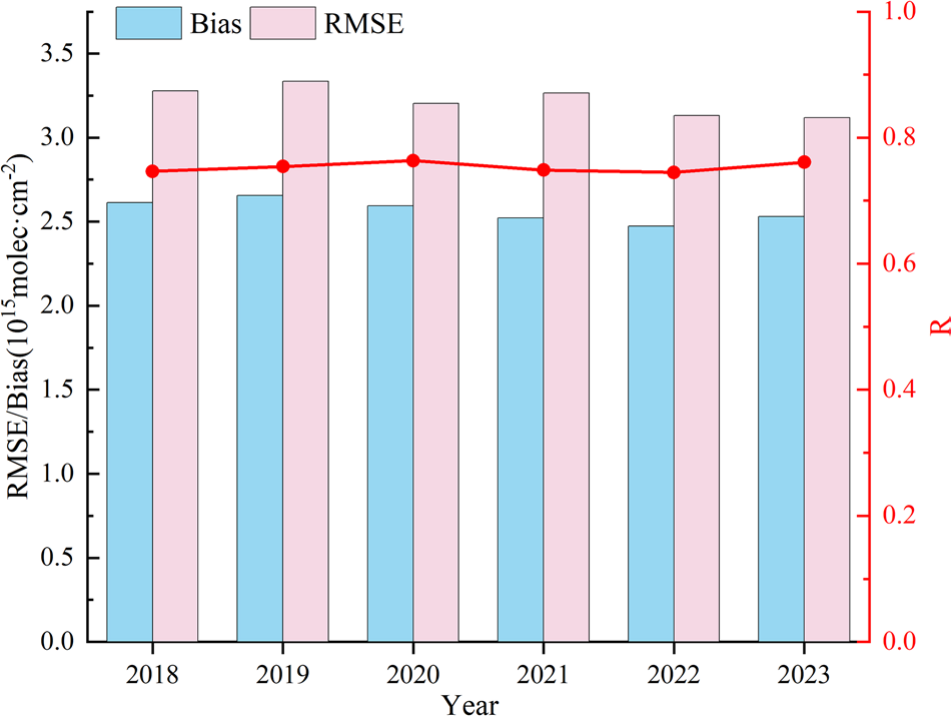
Assessment of the oversampling dataset against the simulation data, with the blue columns representing the annual mean of the bias, the pink columns indicating the annual mean of the RMSE, and the annual mean of *R* is connected by a red line.

We performed a correlation analysis and consistency test for these two datasets at global scale during the simulation time (Fig. 9). Correlation between the oversampling dataset and the GEOS-Chem simulated data over the past five years is significant, with an average correlation of 0.75. The Bias and RMSE are higher than the comparison with OMHCHOms, with an average bias of 2.56 × 10¹⁵ molec/cm² and an average RMSE of 3.23 × 10¹⁵ molec/cm², indicating slightly larger deviation between the two datasets compared to OMHCHOms.

## Usage Notes

A grided global OMI HCHO Level 3 oversampling dataset, OMHCHOS V1.0, was developed using a self-designed oversampling algorithm. Compared with the products released by EARTHDATA, this dataset provides 7 distinct spatial resolutions and 12 temporal resolutions, thereby enhancing the diversity of HCHO datasets available to users. The highest spatial resolution has been upgraded to 0.05° (~5 km), supporting tracking HCHO emissions at kilometer scale. In addition, OMHCHOS quantitatively tracks the propagation of uncertainty throughout the oversampling algorithm, substantially reducing the uncertainty level of the original data and ensuring a reliable overall data quality. According to the SAO, the spectral fitting process introduces an uncertainty of 40–100%, while the atmospheric mass factor contributes an additional ~30%, resulting in a comprehensive uncertainty of 50–105% for the original OMI L2 HCHO data. By contrast, analysis of OMHCHOS shows a pronounced reduction in relative uncertainty, with a multi-year global mean of only 19%, demonstrating a significant improvement in data accuracy compared with the original L2 product. Furthermore, the dataset achieves near-global coverage, with only minor gaps remaining over the polar regions, and provides flexible options across multiple spatial and temporal scales. High-resolution data enable the identification of kilometer-scale emission sources and support regional-scale emission assessments, whereas coarser spatial resolutions are more suitable for large-scale or global analyses. Similarly, high temporal resolutions facilitate investigations of short-term emission transport and dispersion, while coarser temporal scales are well suited for long-term source–sink evaluations.

**Fig. 10:**
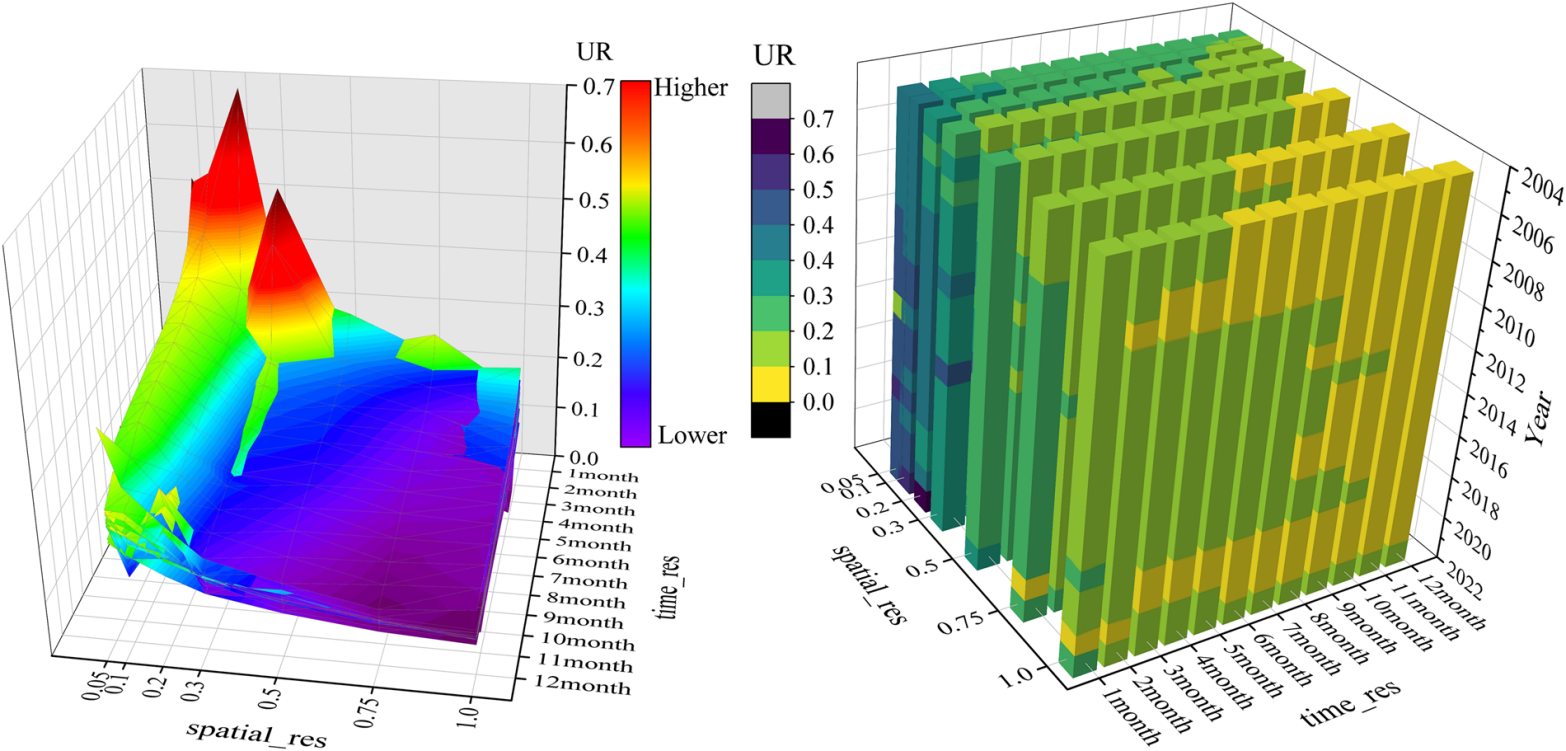
OSTSOM (left) and data cube (right).

Based on OMHCHOS V1.0, an oversampling spatio-temporal scale optimization model (OSTSOM) was established for enabling users to identify the required data efficiently. We assessed the goodness-of-fit for the points of each year and found that the indices for 2007, 2011, 2014 and 2021 were 0.78, 0.71, 0.65 and 0.75, respectively, which were lower than those of other years (>0.9), but the overall goodness-of-fit indices still reached a high level. OSTSOM is shown in Fig. 10 and presents some features: (I) A pronounced aggregation of high UR values is observed. Finer spatial resolution and shorter temporal resolution give higher relative uncertainty. As shown in the Fig. 10, the red high-value regions are concentrated in the range of 0.05°–0.5° for spatial resolution and 1–3 months for temporal resolution, while coarser spatial resolution and longer temporal resolution are associated with lower relative uncertainty, with the purple low-value regions predominantly distributed within the 0.5°–1° spatial resolution and 2–12 months temporal resolution. (II) When the spatial resolution exceeds 0.5°, UR remains consistently low (generally <0.2) and exhibits weak sensitivity to temporal resolution. However, for spatial resolution finer than 0.5°, ER is generally higher (>0.2) and represents increasingly influenced by temporal resolution.

We developed a data lookup table (Table 2) for OSTSOM to foster efficient data selection, enabling users to quickly determine the optimal data in line with their requirements. The lookup table provides two selection methods: the threshold-based division method and the value-domain division method. The data selection principle of the lookup table is to first determine the ideal UR range, and then the appropriate data set in accordance with the maximum frequency of occurrence of SR and TR, TR ≥5% and SR ≥10% of the data points is recommended (see Table S1-S4 for the specific allocation of the percentage of the data points). Users can utilize the lookup table to select data according to their needs. For instance, if UR <0.1 is required, it is recommended to download data with SR ≥0.75° and TR ≥5 months to ensure optimal accuracy and reliability.

**Table 2:** Look-up table recording two division methods.

classification	UR	SR (°)	TR (month)
Threshold-based division method	<0.1	≥0.75	≥5
	<0.2	≥0.3	≥2
	<0.3	≥0.05	≥1
	<0.4	≥0.05	≥1
	<0.5	≥ 0.05	≥1
Value-domain division method	0~0.1	≥0.75	≥5
	0.1~0.2	≥0.2	≥1
	0.2~0.3	≤0.3	≥1
	0.3~0.4	≤0.3	≤5
	0.4~0.5	≤0.1	≤2

We recommend the following data selection strategies: (I) For large-scale or global studies, if minimizing UR is a priority, it is advisable to use coarse SR and longer TR; (II) For regional-scale analyses, if high SR is required, strict control of UR may need to be relaxed. For example, if expecting SR to be as fine as 0.05°, it is a good idea to select the data with UR >0.2; (III) If focusing on smaller TR, it is better to choose data with coarse SR, under the premise of determining TR, the coarser the SR, the better the UR control; (IV) When UR falls within the range of 0.1 to 0.3, TR has minimal impact on data quality, allowing flexibility in SR choices.

We futher constructed a multidimensional data cube to capture the characteristics of the dataset, with temporal resolution as the X-axis, spatial resolution as the Y-axis, and year as the Z-axis. The yearly average UR is represented as points within the data cube (Fig. 10). Users can locate target data efficiently by consulting the oversampling spatial-temporal scale optimization model and look-up tables. This data cube focuses on outliers and trends within the dataset across years. For instance, when selecting data at resolution of 0.05°, the cube immediately reports that the UR of 2014 is higher than that of other years, aligning with the low goodness-of-fit index for 2014 in the production of OSTSOM. The four-dimensional oversampling data cube also tells users that data points exhibiting clear anomalies have been excluded in advance, primarily those with spatial resolutions of 0.1° and 0.2° from the years 2006 and 2007. Data with a UR of more than 0.5 (unideal data) are labeled in Table 3. It also shows the year and spatial resolution of the unideal data, while the values in the table represent the temporal resolution at which the dissatisfaction occurred, which can assist users in locating the unideal data quickly, so as to avoid the disturbance to data analysis caused by the high uncertainty. The ‘/’ symbol in the Table 3 indicates no anomalies were detected, confirming its reliability for general use.

**Table 3:** Lookup table for unideal data with UR > 0.5.

Year	0.05	0.1	0.2	0.3	0.5	0.75	1
SR							
2005	/	/	/	/	/	/	/
2006	/	/	3–12	/	/	/	/
2007	/	4–12	/	/	/	/	/
2008	/	/	/	/	/	/	/
2009	/	/	/	/	/	/	/
2010	/	/	/	/	/	/	/
2011	/	/	/	/	/	/	/
2012	1	/	/	/	/	/	/
2013	/	/	/	/	8	/	/
2014	/	/	/	/	/	/	/
2015	/	/	/	/	/	/	/
2016	/	/	/	/	/	/	/
2017	/	/	/	/	/	/	/
2018	1	/	/	/	/	/	/
2019	/	/	/	/	/	/	/
2020	/	/	/	/	/	/	/
2021	1	/	/	/	/	/	/
2022	1	1	1	/	/	/	/

## Supplementary information

Supplementary Information

## Data Availability

The OMHCHOS V1.0 dataset is publicly available on the Science Data Bank and can be accessed via the DOI link: 10.57760/sciencedb.29626. It may also be retrieved by entering the full dataset title, “Global OMI HCHO Level 3 Oversampling Dataset”, in the ScienceDB search bar.

## Code availability

Codes for producing this dataset were organized using Fortran 90, R 4.1.2 and Python 3.0, compatible with programming languages at newer version. Codes are available in 10.57760/sciencedb.29626.
