# Supplementary material for: Global OMI HCHO Level-3 oversampling dataset: high spatial resolution and lightweight uncertainty
Source: Sci Data. 2026 Jan 19;13:253. doi: 10.1038/s41597-026-06577-w (PMC12917192; doi:10.1038/s41597-026-06577-w)
Supplement: Supplementary file 1 — Supplementary Information [file 41597_2026_6577_MOESM1_ESM.docx]

Supplementary Information

**Global OMI HCHO Level 3 oversampling dataset: high spatial resolution and lightweight uncertainty**

**Hui Xia^1,2^, Dakang Wang^1,2*^, Xiankun Yang^1,2^, Xicheng Li^3^, Lei Zhu^3^, Tianyu Lu^4^, Zhaolong Song^1,2^, Yongru Mo^1,2^, Chenglong Yan^1,2^, Dongchuan Pu^5^, Xiaoxing Zuo^6.7^, Wenfu Sun^8^, Jinnian Wang^1,2^, Xingfa Gu^1,2^**

^1^School of Geography and Remote Sensing, Guangzhou University, Guangzhou 510006, China

^2^Institute of Aerospace Remote Sensing Innovations, Guangzhou University, Guangzhou 510006, China

^3^School of Environmental Science and Engineering, Southern University of Science and Technology, Shenzhen 518055, China.

^4^College of Science, Northeastern University, Boston, MA02115, United States

^5^School of Architecture & Urban Planning, Shenzhen University, Shenzhen 518060, China

^6^Royal Netherlands Meteorological Institute (KNMI), De Bilt, the Netherlands

^7^Department of Geoscience & Remote Sensing, Delft University of Technology (TUD), Delft, the Netherlands

^8^Division of Atmospheric Composition, Royal Belgian Institute for Space Aeronomy (BIRAIASB), Brussels 1180, Belgium

**Table S1.** Spatial resolution based on the threshold-based division method selection (≥10%)

| UR  SR | 0~0.1 | 0.1~0.2 | 0.2~0.3 | 0.3~0.4 | 0.4~0.5 |
| --- | --- | --- | --- | --- | --- |
| 1 | 0.58 | 0.12 | 0.00 | 0.00 | 0.00 |
| 0.75 | 0.33 | 0.20 | 0.01 | 0.00 | 0.00 |
| 0.5 | 0.07 | 0.28 | 0.04 | 0.00 | 0.00 |
| 0.3 | 0.00 | 0.23 | 0.11 | 0.15 | 0.14 |
| 0.2 | 0.00 | 0.10 | 0.27 | 0.18 | 0.03 |
| 0.1 | 0.00 | 0.06 | 0.26 | 0.30 | 0.44 |
| 0.05 | 0.02 | 0.02 | 0.31 | 0.37 | 0.39 |

**Note:** The yellow markers in the table are the features of the dataset that account for ≥10% of the data set

**Table S2.** Time resolution based on the threshold-based division method selection (≥5%)

| UR TR | 0~0.1 | 0.1~0.2 | 0.2~0.3 | 0.3~0.4 | 0.4~0.5 |
| --- | --- | --- | --- | --- | --- |
| 1month | 0.01 | 0.05 | 0.06 | 0.25 | 0.81 |
| 2month | 0.02 | 0.08 | 0.06 | 0.26 | 0.11 |
| 3month | 0.03 | 0.08 | 0.09 | 0.18 | 0.03 |
| 4month | 0.04 | 0.08 | 0.11 | 0.09 | 0.00 |
| 5month | 0.05 | 0.09 | 0.11 | 0.05 | 0.00 |
| 6month | 0.07 | 0.09 | 0.10 | 0.03 | 0.00 |
| 7month | 0.08 | 0.09 | 0.10 | 0.03 | 0.00 |
| 8month | 0.10 | 0.09 | 0.09 | 0.03 | 0.00 |
| 9month | 0.14 | 0.08 | 0.09 | 0.03 | 0.00 |
| 10month | 0.16 | 0.08 | 0.08 | 0.03 | 0.00 |
| 11month | 0.15 | 0.09 | 0.06 | 0.01 | 0.03 |
| 12month | 0.16 | 0.09 | 0.06 | 0.01 | 0.03 |

**Note:** The yellow markers in the table are the features of the dataset that account for ≥5% of the data set

**Table S3.** Spatial resolution based on the value-domain division method selection (≥10%)

| UR  SR | ＜0.1 | ＜0.2 | ＜0.3 | ＜0.4 | ＜0.5 |
| --- | --- | --- | --- | --- | --- |
| 1 | 0.58 | 0.24 | 0.17 | 0.15 | 0.14 |
| 0.75 | 0.33 | 0.24 | 0.17 | 0.15 | 0.14 |
| 0.5 | 0.07 | 0.22 | 0.16 | 0.15 | 0.14 |
| 0.3 | 0.00 | 0.16 | 0.15 | 0.15 | 0.15 |
| 0.2 | 0.00 | 0.07 | 0.14 | 0.14 | 0.14 |
| 0.1 | 0.00 | 0.04 | 0.11 | 0.13 | 0.14 |
| 0.05 | 0.02 | 0.02 | 0.11 | 0.14 | 0.14 |

**Note:** The yellow markers in the table are the features of the dataset that account for ≥10% of the data set

**Table S4.** Time resolution based on the value-domain division method selection (≥5%)

| UR  TR | ＜0.1 | ＜0.2 | ＜0.3 | ＜0.4 | ＜0.5 |
| --- | --- | --- | --- | --- | --- |
| 1month | 0.01 | 0.04 | 0.05 | 0.07 | 0.08 |
| 2month | 0.02 | 0.06 | 0.06 | 0.08 | 0.08 |
| 3month | 0.03 | 0.07 | 0.07 | 0.09 | 0.08 |
| 4month | 0.04 | 0.07 | 0.08 | 0.09 | 0.08 |
| 5month | 0.05 | 0.08 | 0.09 | 0.09 | 0.08 |
| 6month | 0.07 | 0.09 | 0.09 | 0.09 | 0.08 |
| 7month | 0.08 | 0.09 | 0.09 | 0.09 | 0.08 |
| 8month | 0.10 | 0.09 | 0.09 | 0.08 | 0.08 |
| 9month | 0.14 | 0.09 | 0.09 | 0.09 | 0.08 |
| 10month | 0.16 | 0.10 | 0.09 | 0.09 | 0.08 |
| 11month | 0.15 | 0.11 | 0.09 | 0.08 | 0.08 |
| 12month | 0.16 | 0.11 | 0.09 | 0.08 | 0.08 |

**Note:** The yellow markers in the table are the features of the dataset that account for ≥5% of the data set
